# Outcomes and risk factors for infection after endovascular treatment in patients with acute ischemic stroke

**DOI:** 10.1111/cns.14753

**Published:** 2024-05-10

**Authors:** Xin Jiang, Yaowen Hu, Jian Wang, Mengmeng Ma, Jiajia Bao, Jinghuan Fang, Li He

**Affiliations:** ^1^ Department of Neurology, West China Hospital Sichuan University Chengdu China

**Keywords:** acute ischemic stroke, clinical outcome, endovascular treatment, infection

## Abstract

**Aims:**

Infection is a common complication following acute ischemic stroke (AIS) and significantly contributes to poor functional outcomes after stroke. This study aimed to investigate the effects of infection after endovascular treatment (post‐EVT infection) on clinical outcomes and risk factors in patients with AIS.

**Methods:**

We retrospectively analyzed AIS patients treated with endovascular treatment (EVT) between January 2016 and December 2022. A post‐EVT infection was defined as any infection diagnosed within 7 days after EVT. The primary outcome was functional independence, defined as a modified Rankin scale (mRS) score of 0–2 at 90 days. A multivariable logistic regression analysis was conducted to determine independent predictors of post‐EVT infection and the associations between post‐EVT infection and clinical outcomes.

**Results:**

A total of 675 patients were included in the analysis; 306 (45.3%) of them had post‐EVT infections. Patients with post‐EVT infection had a lower rate of functional independence than patients without infection (31% vs 65%, *p* = 0.006). In addition, patients with post‐EVT infection achieved less early neurological improvement (ENI) after EVT (25.8% vs 47.4%, *p* < 0.001). For safety outcomes, the infection group had a higher incidence of any intracranial hemorrhage (23.9% vs 15.7%, *p* = 0.01) and symptomatic intracranial hemorrhage (10.1% vs 5.1%, *p* = 0.01). Unsuccessful recanalization (aOR 1.87, 95% CI 1.11–3.13; *p* = 0.02) and general anesthesia (aOR 2.22, 95% CI 1.25–3.95; *p* = 0.01) were identified as independent predictors for post‐EVT infection in logistic regression analysis.

**Conclusion:**

AIS patients who develop post‐EVT infections are more likely to experience poor clinical outcomes. Unsuccessful recanalization and general anesthesia were independent risk factors for the development of post‐EVT infection.

## Graphical abstract

Post‐EVT infection was associated with poor clinical outcomes in patients with acute ischemic stroke.

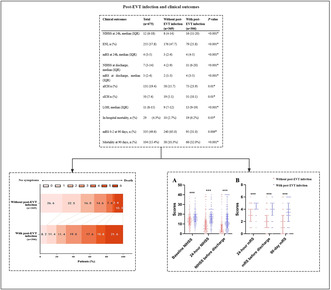

## 1 INTRODUCTION

Endovascular treatment (EVT) has been proven to be the standard reperfusion therapy for patients with acute ischemic stroke due to large vessel occlusion (LVO), which can dramatically reduce disability and mortality.^1^, ^2^, ^3^ A positive clinical effect of EVT has also been observed in real‐world practice, even beyond randomized trials.^4^, ^5^ Nevertheless, despite successful recanalization, more than half of patients who underwent EVT did not regain functional independence.^6^ It is still necessary to explore other key determinants of poor recovery despite successful reperfusion after EVT to further improve the clinical outcomes for AIS.

Infection is a common complication of stroke and a major cause of poor functional outcomes after stroke.^7^, ^8^ In contrast to the overwhelming clinical efficacy of EVT, little attention has been paid to the potential impact of complications after EVT. EVT often requires general anesthesia, and the procedure time is determined by the condition of the occluded vessel.^9^ As a result, EVT may be accompanied by a variety of intraoperative and postoperative complications, including infection, which may lead to prolonged invasive ventilation and intensive care.^10^ Prospective observational studies showed that patients who received EVT had a significantly higher risk of pneumonia than those who received medical treatment alone.^11^ Additionally, high baseline white blood cell count, neutrophil count, and neutrophil to lymphocyte ratio are reported to be independently associated with poor functional outcome, a high incidence of symptomatic intracranial hemorrhage (sICH), and mortality after EVT, suggesting that infection after EVT may affect the clinical outcome to some extent.^12^, ^13^, ^14^ Our previous studies indicated that fever after EVT (body temperature >38°C) was significantly correlated with early neurological deterioration and poor functional prognosis at 90 days, and univariate analysis suggested that patients with poor prognosis had a higher incidence of infection, further supporting the association between postoperative infection and poor prognosis.^15^ Existing studies have mainly focused on the relationship between stroke‐associated infection (SAI) and clinical outcomes, but no study has explored the relationship between infection after EVT and prognosis. Therefore, our study intends to investigate the effects of post‐EVT infection on clinical outcomes in patients with AIS and to further explore risk factors for post‐EVT infection.

## 2 METHODS

### 2.1 Study participants and evaluation

All consecutive patients who underwent EVT between January 2016 and December 2022 in West China Hospital were retrospectively retrieved. Patients were selected if they: (1) were aged 18 years or over; (2) diagnosed with acute ischemic stroke; (3) caused by large vessel occlusion confirmed by computed tomographic angiography (CTA), magnetic resonance angiography (MRA), or digital subtraction angiography (DSA); (4) time from stroke onset to groin puncture within 24 h; (5) had a premorbid modified Rankin scale (mRS) score <2; (6) received EVT; and (7) no infection was diagnosed before EVT, including pneumonia, urinary tract infection (UTI), and septicemia. This study was performed in accordance with the ethical principles of the 1964 Declaration of Helsinki and its later amendments and approved by the Ethics Committee of West China Hospital [No. 2020(69)]. The need to obtain written informed consent was waived because of the retrospective and observational nature of the study.

Post‐EVT infection was defined as any infection diagnosed within 7 days after EVT, including pneumonia, urinary tract infection, and septicemia. Infection was diagnosed based on the modified Centers for Disease Control and Prevention (CDC) criteria by trained and experienced clinicians and was divided into three groups: pneumonia, UTI, and septicemia.^16^ The clinical diagnosis of pneumonia was based on the following findings: a new or progressive infiltrate, consolidation, or ground glass opacity revealed on chest computed tomography (CT) or radiography, plus two or more of the following three criteria: (1) fever (>38°C) without another cause; (2) leukopenia (<4000 leukocytes/mm^3^) or leukocytosis (>10,000 leukocytes/mm^3^); and (3) for patients older than 70 years old, at least two of the following: (a) a positive sputum culture; (b) a new onset or worsening cough or respiratory rate; (c) rales, crackles, or bronchial breath sounds; and (d) worsening gas exchange.^17^, ^18^ UTI was defined based on the presence of relevant clinical symptoms and/or signs (e.g., dysuria and changes in urinary frequency) with positive microbiological cultures, negative cultures with leukocytosis, fever (temperature ≥38°C), or both.^19^ Septicemia was defined as: (1) at least one of the following signs or symptoms: fever (>38°C), chills, or hypotension; and (2) positive blood cultures (common skin‐contaminating bacteria such as *Staphylococcus epidermidis* need to be isolated from 2 or more blood cultures).^20^

### 2.2 Data collection and outcome measures

Individual patient data with baseline characteristics and clinical information, including age, sex, medical history, baseline blood pressure, baseline Alberta Stroke Program Early Computed Tomography Score (ASPECTS) score, admission National Institutes of Health Stroke Scale (NIHSS), results of pre‐EVT laboratory examination, treatment with intravenous thrombolysis, site of intracranial vessel occlusion, procedure of EVT, time to groin puncture, length of hospital stay, and modified Thrombolysis in Cerebral Infarction (mTICI) score.

The 3‐month mRS score was used as a graded interval scale (range from 0 points indicating no symptoms to 6 points indicating death) for the evaluation of neurological functional disability.^21^ The primary outcome was functional independence, which was defined as mRS 0–2. The secondary outcome measures were as follows: early neurological improvement (ENI), which was defined as a decrease of 4 points or more in NIHSS score at 24 h compared with admission; NIHSS score at 24 h after EVT; mRS score at 24 h after EVT; NIHSS score at discharge; mRS score at discharge; any intracranial hemorrhage (aICH), sICH; in‐hospital mortality and mortality at 90 days, which was defined as a mRS score of 6; and the length of hospital stay.^22^

### 2.3 Statistical analysis

Continuous variables with a normal distribution were described as the mean with standard deviation (SD) or median with interquartile range (IQR) if not normally distributed. Categorical variables were expressed as counts (%). Continuous variables were compared using the t test (normal distribution) and the Mann–Whitney *U* test (non‐normal distribution), and categorical variables were compared using the chi‐square test or Fisher's exact test. A multivariable logistic regression analysis was conducted to determine independent predictors of post‐EVT infection. The variables with a *p* value <0.1 in the univariate analyses and clinically relevant factors were included in the logistic regression analysis, with entry and removal limits set at 0.05 and 0.1, respectively, using the stepwise method. In addition, *p* < 0.05 was considered statistically significant, and all data were analyzed with IBM SPSS 26.0 and GraphPad Prism 8.0.

## 3 RESULTS

### 3.1 Demographic and clinical characteristics

**FIGURE 1:**
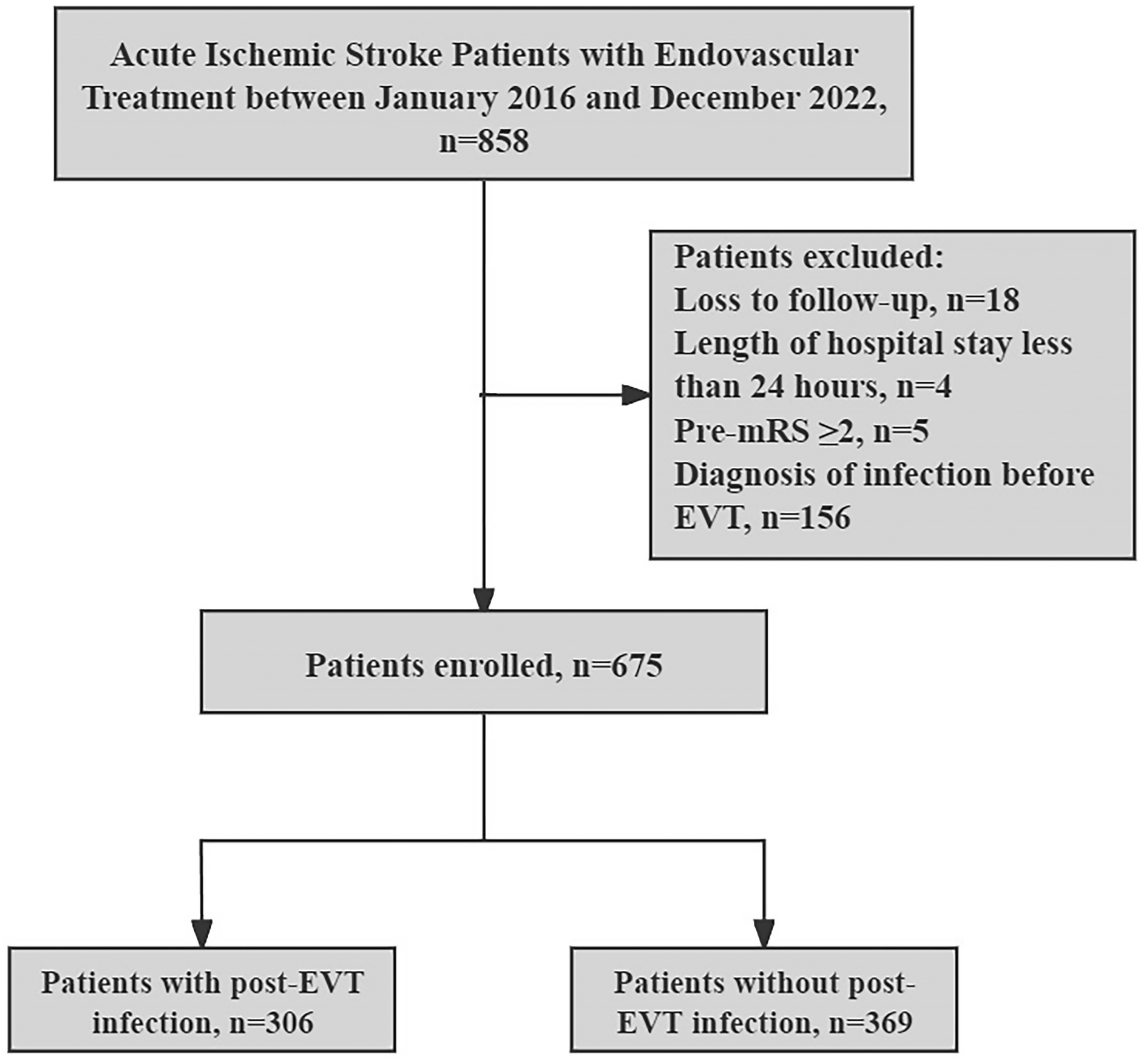
Flowchart of patient enrollment.

Participant enrollment is shown in Figure 1. A total of 675 patients with a median age of 69 years were included in the analysis (Table 1). Among all the included patients, 55.3% were men, 23.4% received intravenous thrombolysis, and the median baseline NIHSS score was 14. There were 306 patients with post‐EVT infection, including 298 patients (97.4%) with pneumonia, 22 patients (7.2%) with urinary tract infection, and 10 patients (3.3%) with septicemia. The median age of patients with post‐EVT infection was 72 years, which was significantly higher than that of patients without post‐EVT infection (66 years old, *p* < 0.001). In addition, patients with post‐EVT infection had a significantly higher baseline systolic blood pressure than those without (146.3 mmHg vs 141.4 mmHg; *p* = 0.02). Moreover, patients with post‐EVT infection had a higher baseline glucose (*p* = 0.04), worse renal function (*p* < 0.001), a higher rate of hypertension (*p* = 0.001), a higher rate of atrial fibrillation (*p* = 0.002), and a lower rate of local anesthesia (*p* = 0.02) than those without post‐EVT infection (Table 1).

**TABLE 1:** Baseline characteristics of study patients.

Variable	Total (*n* = 675)	Without post‐EVT infection (*n* = 369)	With post‐EVT infection (*n* = 306)	*p* value
Age, median (IQR)	69 (57–77)	66 (55–75)	72 (61–80)	<0.001*
Male, *n* (%)	373 (55.3)	214 (58.0)	159 (52.0)	0.12
Smoking, *n* (%)	215 (31.9)	120 (32.5)	95 (31.0)	0.68
DM, *n* (%)	112 (16.6)	53 (14.4)	59 (19.3)	0.09
Hypertension, *n* (%)	361 (53.5)	175 (47.4)	186 (60.8)	0.001*
Pre‐stroke, *n* (%)	78 (11.6)	36 (9.8)	42 (13.7)	0.11
SBP, mea*n* (SD)	143.6 (25.8)	141.4 (24.8)	146.3 (26.7)	0.02*
DBP, mea*n* (IQR)	85 (74–96)	85 (73–95)	85 (75–96)	0.50
AF, *n* (%)	290 (43.0)	139 (37.7)	151 (49.3)	0.002*
Glu, median (IQR)	7.2 (6.2–8.6)	7.0 (6.2–8.4)	7.3 (6.3–9.3)	0.04*
eGFR, median (IQR)	86.4 (69.8–97.0)	89.3 (76.3–99.1)	86.7 (65.0–94.7)	<0.001*
UA, median (IQR)	339 (277.0–416.0)	341.0 (277.0–408.5)	338.5 (278.8–430.0)	0.37
TC, median (IQR)	4.1 (3.5–4.8)	4.1 (3.5–4.9)	4.1 (3.5–4.8)	0.48
TG, median (IQR)	1.2 (0.9–1.7)	1.2 (0.9–1.7)	1.1 (0.9–1.6)	0.77
LDL, median (IQR)	2.5 (1.9–3.1)	2.5 (1.9–3.1)	2.4 (1.8–3.1)	0.49
HDL, median (IQR)	1.2 (1.0–1.5)	1.2 (1.0–1.5)	1.2 (1.0–1.5)	0.83
ASPECTS mean (IQR)	8 (7–9)	8 (6–9)	8 (7–9)	0.76
Baseline NIHSS, median (IQR)	14 (11–18)	13 (9–17)	16 (12–20)	<0.001*
Intravenous rt‐PA, *n* (%)	158 (23.4)	82 (22.2)	76 (24.8)	0.43
Occlusion site				0.52
ICA, *n* (%)	206 (30.5)	103 (27.9)	103 (33.7)	
MCA, *n* (%)	388 (57.5)	214 (58.0)	174 (56.9)	
ACA, *n* (%)	31 (4.6)	19 (5.1)	12 (3.9)	
PCA, *n* (%)	8 (1.2)	5 (1.4)	3 (1.0)	
BA, *n* (%)	79 (11.7)	48 (13.0)	31 (10.1)	
VA, *n* (%)	21 (3.1)	10 (2.7)	11 (3.6)	
OTP, median (IQR)	295.0 (224.8–401.3)	295.0 (222.0–408)	295.0 (226.0–390.0)	0.80
OTR, median (IQR)	375.0 (297.0–494.3)	370.0 (292.0–505.0)	379.0 (300.0–490.0)	0.79
PTR, median (IQR)	70 (45–101)	70 (45–100)	71 (50–105)	0.19
Local anesthesia, *n* (%)	106 (15.7)	69 (18.7)	37 (12.1)	0.02*
Stent retriever, *n* (%)	424 (62.8)	215 (58.3)	209 (68.3)	0.01*
Aspiration, *n* (%)	385 (57.0)	213 (57.7)	172 (56.2)	0.69
Successful recanalization, *n* (%)	583 (86.4)	327 (88.6)	256 (83.7)	0.06

Abbreviations: ACA, anterior cerebral artery; AF, atrial fibrillation; ASPECTS, the Alberta Stroke Program Early CT Score; BA, basilar artery; DBP, diastolic blood pressure; DM, diabetes mellitus; eGFR, estimated Glomerular Filtration Rate; Glu, serum glucose; HDL, high‐density lipoprotein; ICA, internal carotid artery; IQR, interquartile range; LDL, low‐density lipoprotein; MCA, middle cerebral artery; NIHSS, National Institutes of Health Stroke Scale; OTP, time from onset to treatment; OTR, time from onset to reperfusion; PCA, posterior cerebral artery; PTR, time from groin puncture to reperfusion; rt‐PA, recombinant tissue plasminogen activator; SBP, systolic blood pressure; SD, standard deviation; TC, total cholesterol; TG, Triglyceride; UA, uric acid; VA, vertebral artery.

*p < 0.05, significant in univariate analysis.

### 3.2 Post‐EVT infection and clinical outcomes

**FIGURE 2:**
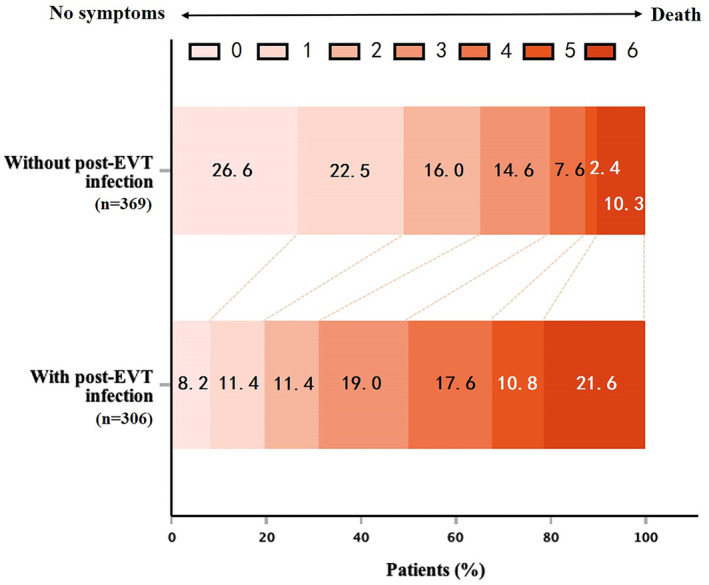
Functional outcome at 3 months in patients with or without post‐EVT infection, assessed using the modified Rankin scale (mRS).

**FIGURE 3:**
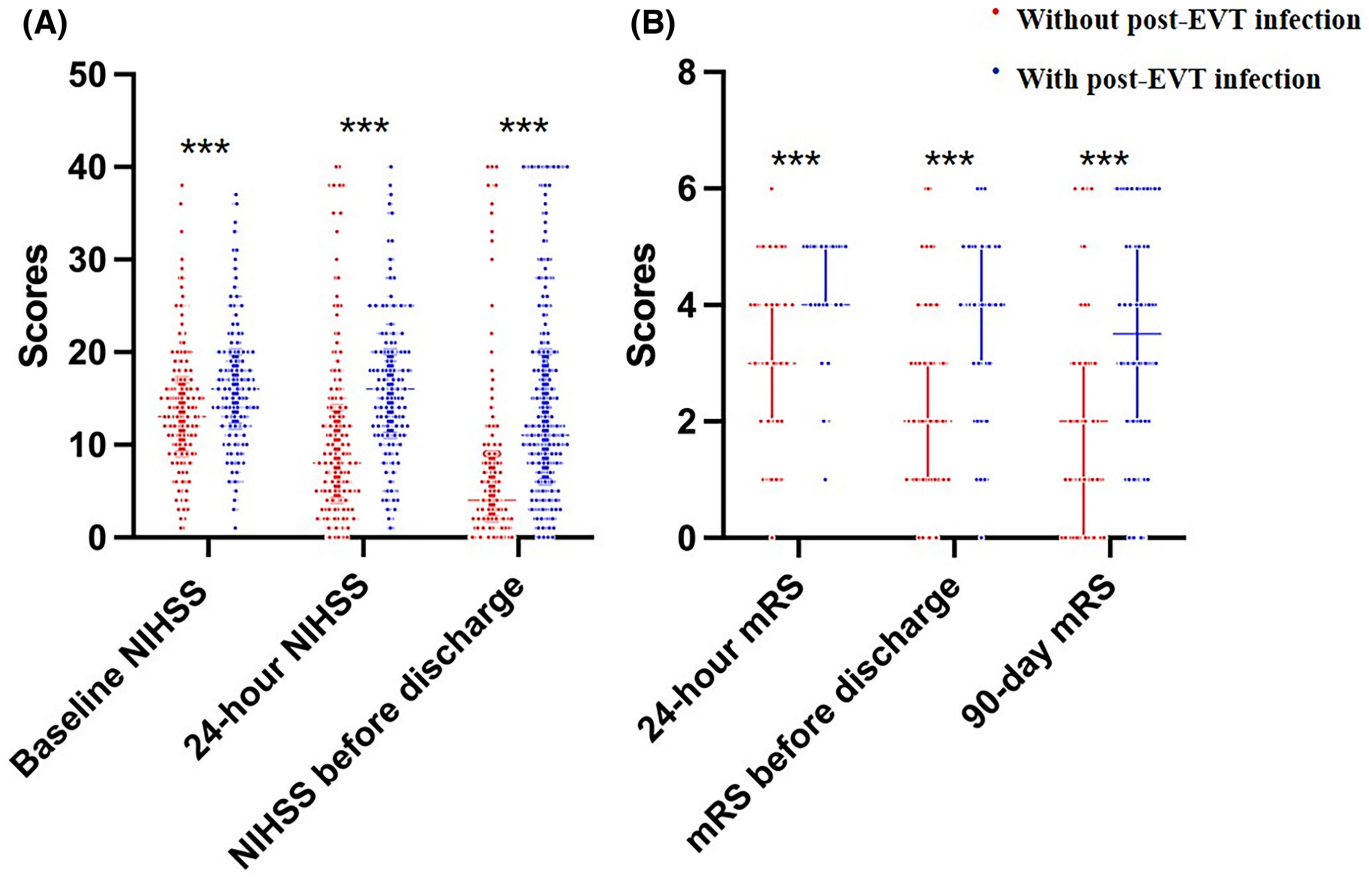
Comparison of NIHSS and mRS Score between the two groups at different time points (A) NIHSS score at admission, 24 h after EVT, and before discharge; (B) mRS score at 24 h after EVT, before discharge, and 90 days after EVT; three asterisks indicate *p* < 0.001).

Our results showed that patients with post‐EVT infection had a lower rate of functional independence than patients without infection (31% vs 65%, *p* = 0.006) (Figure 2). In addition, patients with post‐EVT infection had a lower rate of ENI (25.8% vs 47.4%, *p* < 0.001). The NIHSS score at 24 hours after EVT (16 vs 8, *p* < 0.001), and mRS score at 24 hours after EVT (4 vs 3, *p* < 0.001), NIHSS score at discharge (11 vs 4, *p* < 0.001), mRS score at discharge (4 vs 2, *p* < 0.001) were all higher in the post‐EVT infection group (Figure 3). For safety outcomes, we found that the post‐EVT infection group had a higher incidence of aICH (23.9% vs 15.7%, *p* = 0.01) and sICH (10.1% vs 5.1%, *p* = 0.01). Furthermore, patients with post‐EVT infection had higher in‐hospital mortality (6.2% vs 2.7%; *p* = 0.03) and mortality at 90 days (32.0% vs 10.3%; *p* < 0.001) (Table 2). The post‐EVT infection group showed a significantly longer length of hospital stay (13 days) than those without (9 days, *p* < 0.001).

**TABLE 2:** Clinical outcomes of study patients.

Clinical outcomes	Total (*n* = 675)	Without post‐EVT infection (*n* = 369)	With post‐EVT infection (*n* = 306)	*p* value
NIHSS at 24 h, median (IQR)	12 (6–18)	8 (4–14)	16 (11–20)	<0.001*
ENI, *n* (%)	255 (37.8)	176 (47.7)	79 (25.8)	<0.001*
mRS at 24 h, median (IQR)	4 (3–5)	3 (2–4)	4 (4–5)	<0.001*
NIHSS at discharge, median (IQR)	7 (3–14)	4 (2–9)	11 (6–20)	<0.001*
mRS at discharge, median (IQR)	3 (2–4)	2 (1–3)	4 (3–5)	<0.001*
aICH, *n* (%)	131 (19.4)	58 (15.7)	73 (23.9)	0.01*
sICH, *n* (%)	50 (7.4)	19 (5.1)	31 (10.1)	0.01*
LOH, median (IQR)	11 (8–15)	9 (7–12)	13 (9–19)	<0.001*
In‐hospital mortality, *n* (%)	29 (4.3%)	10 (2.7%)	19 (6.2%)	0.03*
mRS 0–2 at 90 days, *n* (%)	335 (49.6)	240 (65.0)	95 (31.0)	0.006*
Mortality at 90 days, *n* (%)	104 (15.4%)	38 (10.3%)	66 (32.0%)	<0.001*

Abbreviations: aICH, any intracerebral hemorrhage; ENI, early neurological improvement; IQR, interquartile range; LOH, length of hospital stay; mRS, modified Rankin Scale; NIHSS, National Institutes of Health Stroke Scale; sICH, symptomatic intracerebral hemorrhage.

*p < 0.05, significant in univariate analysis.

### 3.3 Multivariate analyses for risk factors and outcomes

After adjusting for age, sex, systolic blood pressure, ASPECTS, glucose, diabetes mellitus, hypertension, estimated glomerular filtration rate, prestroke history, atrial fibrillation, time from groin puncture to reperfusion, unsuccessful recanalization, and general anesthesia, the independent predictors of post‐EVT infection were unsuccessful recanalization (aOR 1.87, 95% CI 1.11–3.13; *p* = 0.02) and general anesthesia (aOR 2.22, 95% CI 1.25–3.95; *p* = 0.01) (Table 3). In addition, the tolerance levels of the variables included in multivariate analyses were all ≥0.1, and the variance inflation factor was less than 10, suggesting that there was no collinearity among the variables. Our results revealed that post‐EVT infection was strongly related to clinical outcomes. Post‐EVT infection was positively associated with 90‐day mortality (aOR 2.04, 95% CI 1.24–3.35, *p* = 0.005) and negatively associated with functional independence (aOR 0.27, 95% CI 0.18–0.41, *p* < 0.001) (Table 4).

**TABLE 3:** Logistic regression analysis of possible risk factors for post‐EVT infection.

Variables	aOR	95% CI	*p* value
Age	1.01	0.99–1.03	0.25
Gender	1.08	0.75–1.57	0.68
SBP	1.00	0.99–1.01	0.49
ASPECTS	0.96	0.88–1.05	0.36
Glu	1.02	0.96–1.10	0.52
DM	0.99	0.98–1.00	0.10
eGFR	1.2	0.72–2.01	0.49
Hypertension	1.26	0.85–1.87	0.25
Pre‐stroke	1.06	0.60–1.87	0.84
AF	1.16	0.79–1.71	0.44
PTR	1.00	0.99–1.01	0.17
Unsuccessful recanalization	1.87	1.11–3.13	0.02*
General anesthesia	2.22	1.25–3.95	0.01*

Abbreviations: AF, atrial fibrillation; aOR, adjusted odds ratio; ASPECTS, the Alberta Stroke Program Early CT Score; CI, confidence interval; DM, diabetes mellitus; eGFR, estimated Glomerular Filtratkon Rate; Glu, serum glucose; PTR, time from groin puncture to reperfusion; SBP, systolic blood pressure.

*p < 0.05, significant in multivariate analysis.

**TABLE 4:** Impacts of post‐EVT infection on clinical outcomes in the multivariate model.

Outcomes	aOR	95% CI	*p* value
In‐hospital mortality	1.60	0.65–3.98	0.31
90 days mortality	2.04	1.24–3.35	0.005*
mRS 0–2	0.27	0.18–0.41	<0.001*

Abbreviations: aOR, adjusted odds ratio; CI, confidence interval; mRS, modified Rankin Scale.

*p < 0.05, significant in multivariate analysis.

## 4 DISCUSSION

Infection has already been considered a determinant of outcomes after stroke; however, existing studies have mainly focused on the relationship between stroke‐associated infection and clinical outcome in patients with AIS.^7^, ^8^, ^23^, ^24^ In our study, we compared and summarized the baseline characteristics, risk factors, and clinical outcomes of AIS patients with or without post‐EVT infection.

Previous studies have reported that the incidence of stroke‐associated infection ranges from 5.4 to 44%, depending on the clinical setting and definition.^25^, ^26^ In our study, 45.3% of the patients with LVO stroke treated with endovascular treatment developed post‐EVT infection, which was slightly higher than in previous studies. Possible reasons can be attributed to the rate of general anesthesia approaching 90% in our study and the failure to achieve recanalization in some of the enrolled patients.

Stroke‐associated infection has been confirmed by studies to be closely related to the poor prognosis of patients with AIS.^8^, ^27^, ^28^ In this study, we proposed the concept of post‐EVT infection for the first time and investigated its association with clinical outcome in patients with AIS who received EVT. Studies have found that patients who received EVT had a higher rate of medical complications, and patients with fever in the early stages after EVT had a worse clinical outcome than those without.^11^, ^15^, ^29^ Our results are consistent with this hypothesis and preliminarily validate the findings of previous studies to some extent. There are several possible reasons for the poor outcome in those with post‐EVT infection. First and foremost, patients with post‐EVT infection may be intubated for longer periods of time, which may require longer intensive care and a longer length of hospital stay, which could delay early rehabilitation. Second, stroke and the procedure of EVT may produce a considerable inflammatory response with both peripheral and central production of proinflammatory cytokines, chemokines, and cell adhesion molecules, thus affecting patient prognosis.^30^, ^31^, ^32^ In addition, our study found a significant difference in age between the two groups, which was consistent with the findings of Muhl et al.^23^ Previous studies have revealed that elderly patients who receive EVT have worse functional outcomes than younger patients.^33^, ^34^ Notably, in our study, 120 patients were aged over 80, with 78 of them experiencing post‐EVT infection. Elderly patients may have a higher risk of post‐EVT infection than young patients, which may reflect a higher incidence of post‐EVT complications in older patients, including impairment of the swallowing and cough reflex.^35^

Our study demonstrated that patients with unsuccessful recanalization and general anesthesia were more likely to develop post‐EVT infection, which was not entirely consistent with the results of previous studies.^25^, ^36^, ^37^, ^38^ Successful recanalization after endovascular treatment has been reported to be closely related to clinical prognosis.^39^, ^40^ Failure of recanalization leads to poor cerebral blood flow revascularization, which may lead to more severe neurological deficits and an altered state of consciousness, thereby contributing to the development of post‐EVT infection.^41^, ^42^, ^43^ In addition, patients with unsuccessful recanalization may require longer periods of mechanical ventilation as well as intensive care after EVT, increasing the risk of post‐EVT infection. In this study, we noticed that general anesthesia was an independent predictor of post‐EVT infection. General anesthesia has been frequently compared with local anesthesia in numerous studies, but the conclusions are inconsistent. Some studies indicated that patients treated with general anesthesia had worse functional outcomes than those without, which was largely consistent with our study.^44^, ^45^ In contrast, some studies have shown that general anesthesia has a comparable or even better influence on clinical outcomes.^46^, ^47^ The effect of general anesthesia on clinical outcomes after EVT has always been a controversial issue. Interestingly, some studies found that general anesthesia was associated with higher recanalization rates.^46^, ^47^ In our study, general anesthesia, together with unsuccessful recanalization, were risk factors for post‐EVT infection, which may be due to the effect of mechanical ventilation and sedation under general anesthesia.^48^, ^49^

To the best of our knowledge, this is the first study that directly evaluates the effects of post‐EVT infection in patients with LVO stroke. Our results have implications for clinical practice and may have a positive influence on the development of post‐EVT infection and clinical trials for AIS. Our study demonstrated that, for AIS patients who underwent EVT, post‐EVT infection was associated with worse functional outcomes, higher rates of intracranial hemorrhage, higher mortality, and a longer length of hospital stay. Our study initially provides a basis for intervention for post‐EVT infection; however, prospective, large sample size studies are needed to further verify this conclusion.

This study also contains some limitations. First, a retrospective observational design was used in the present study. Therefore, we could not conclude a causal relationship between post‐EVT infection and clinical outcome based on the present results. Second, we could not extract complete data on dysphagia due to the retrospective study design, so we did not include dysphagia in the analysis, which may have some confounding bias on the results. Third, we did not perform subgroup analyses of patients who received prophylactic antibiotics after EVT; further studies are needed to explore the effect of prophylactic antibiotic use. Fourth, in our study, we enrolled patients who underwent EVT from 2016 to 2022, and the rate of successful recanalization has increased with recent advances in thrombectomy technology. A longer study period, changes in the successful recanalization rate, and improvements in medical care may influence the results. Finally, follow‐up data were missing for approximately 2% of the patients, which was a relatively low rate of loss to follow‐up, and we imputed 3% missing values in data by mode in ASPECTS, which may introduce some bias on mean and deviation.

## 5 CONCLUSION

Post‐EVT infection is a major clinical problem for patients with AIS that has a strong independent and negative effect on clinical outcome. Unsuccessful recanalization and general anesthesia were related to the post‐EVT infection. Clinicians should pay attention to post‐EVT infection, and corresponding interventions are needed to improve the clinical prognosis for patients who underwent EVT. Further studies are needed to improve our basic understanding of how and why post‐EVT infection occur and to develop appropriate strategies to prevent or mitigate the deleterious effects of post‐EVT infection on clinical outcomes.

## AUTHOR CONTRIBUTIONS

XJ: Conceptualization; writing–original draft; writing–review and editing; methodology; formal analysis; software; investigation; data curation. YH: Conceptualization; writing–original draft; writing–review and editing; methodology; formal analysis; investigation. JW: Formal analysis; data curation; methodology. MM: data curation; methodology. JB: data curation; methodology. JF: Conceptualization; methodology; software; data curation; investigation; formal analysis; supervision; visualization; writing–original draft; writing–review and editing. LH: Conceptualization; methodology; software; data curation; investigation; formal analysis; supervision; visualization; writing– original draft; writing–review and editing; project administration; resources.

## FUNDING INFORMATION

This study was supported by the Science and Technology Planning Project of Sichuan Province (No. 2021YJ0437) and the National Natural Science Foundation of China (No. 82101395).

## CONFLICT OF INTEREST STATEMENT

The authors declare no competing interests.

## INSTITUTIONAL REVIEW BOARD STATEMENT

This study was performed in accordance with the ethical principles of the 1964 Declaration of Helsinki and approved by the Ethics Committee of West China Hospital [No.2020(69)].

## PATIENT CONSENT FOR PUBLICATION

Not applicable.

## Data Availability

The clinical data used in this study can be obtained from the corresponding authors upon reasonable request.
